# Exploring hiPSC-CM replacement therapy in ischemic hearts

**DOI:** 10.1007/s00395-025-01117-w

**Published:** 2025-06-10

**Authors:** Giuseppe Cipriano, Thomas Thum, Natalie Weber

**Affiliations:** 1 Institute of Molecular and Translational Therapeutic Strategies (IMTTS), Hannover Medical School, 30625 Hannover, Germany; 2 Hannover Medical School, Germany, Dean’s Office for Academic Career Development, nextGENERATION Medical Scientist Program, Hannover, Germany

**Keywords:** Cell therapy, Human induced pluripotent stem cell-derived cardiomyocytes, Ischemic heart disease

## Abstract

Ischemic heart disease is one of the leading causes of heart failure and death worldwide. The loss of cardiomyocytes following a myocardial infarction drives the remodeling process, which, in most cases, ultimately leads to heart failure. Since the available treatment options only slow down the remodeling process without tackling the causes of heart failure onset (i.e., cardiomyocyte loss and inability of the remaining cardiomyocytes to enter the cell cycle and regenerate the heart), in the last two decades, cardiovascular research focused on finding alternative solutions to regenerate the heart. So far, the investigated approaches include a variety of methods aiming at manipulation of non-coding RNAs, such as long non-coding RNA (lncRNA), circular RNA (circRNA), and microRNA (miRNA), and growth factors to enable the cardiomyocytes to re-enter the cell cycle, direct reprogramming of fibroblasts into cardiomyocytes (CM), and CM replacement therapy, all of them with the main goal to replace the loss of cardiomyocytes and restore the heart function. The development of reprogramming protocols from somatic cells to induced pluripotent stem cells (iPSCs) by Yamanaka and Takahashi, along with advancements in differentiation protocols to generate almost pure populations of induced pluripotent stem cell-derived cardiomyocytes (iPSC-CMs), has fostered optimism in cardiac regenerative medicine. Despite these advancements, critical concerns arose regarding the survival and retention of the engrafted cells, arrhythmogenicity, and immune response. Over time, much effort has been put into enhancing iPSC-CM therapy with different methods, ranging from anti-apoptotic small molecule-based approaches to tissue engineering. In this review, we discuss the evolution of cardiac cell therapy, highlighting recent advancements and the remaining challenges that must be overcome to translate this promising approach into clinical practice.

## Introduction

Ischemic heart disease (IHD) remains the main risk factor for developing heart failure (HF) and is one of the leading causes of morbidity, hospitalization, and deaths worldwide [50, 88]. As recently outlined in a comprehensive overview [28], the burden of IHD and its progression to HF remains a critical global health issue. The lack of regenerative abilities in the human adult heart results in development of fibrotic scar tissue after myocardial infarction replacing damaged cardiomyocytes. By influencing the tissue stiffness, such a scar prevents the heart from being filled with blood or pumping properly, leading to remodeling and ultimately to heart failure [74, 88]. Impaired coronary blood flow plays a key role in both initiating and sustaining myocardial dysfunction, acting as both a cause and consequence of heart failure progression [27]. Currently, available pharmacological treatments for HF, including angiotensin receptor-neprilysin inhibitors (ARNIs), β-blockers, mineralocorticoid receptor antagonists, and angiotensin-converting enzyme (ACE) inhibitors, slow the remodeling process but do not provide a treatment of the underlying cause acting more symptomatic than curative options [60]. In advanced stages of HF, the only treatment options available are the implantation of a left ventricular assist device (LVAD) and heart transplantation. Despite its “bridge-to-transplant” indication, LVAD implantations are a big advancement in the field, leading to an increased survival rate after 1 and 2 years post-implantation [44]. However, adverse events, such as rehospitalization, stroke, and multi-system organ failure, are frequent [23]. The only solution up to date able to revert the critical condition of HF is heart transplantation. However, the shortage of organs as well as the inability of some patients to receive a transplantation [90] make it an unrealistic standard procedure and require alternative methods.

Given cardiomyocytes' (CMs) limited ability to proliferate postnatally and the limitations in the current state of the art in clinical practice, new approaches are needed to replace CM loss. For these reasons, cardiac research has focused on developing different approaches for heart regeneration in recent decades. These approaches can be generally divided into two main categories: cell-free and cell-based methods [26].

The first category includes all methods using small molecules or transcription factors to induce direct reprogramming of fibroblasts into cardiomyocytes [32, 84], or the methods that induce endogenous cardiomyocyte proliferation and increase their survival. Micro-RNA (miRNA) [2, 13, 17], long non-coding RNA (lncRNA) [51, 89], circular RNA (circRNA) [2, 29], growth factors like VEGF [24, 94] delivered as modified mRNA (modRNA) [94] and extracellular vesicles [6, 20] showed promising results in the field.

On the other hand, cell-based therapies aim to replace cardiomyocyte loss with cardiomyocytes or cardiac progenitors (CPCs) to provide electro-mechanical support, or with cells whose secretome can reverse the remodeling processes of the damaged myocardium such as mesenchymal stem cells (already reviewed here [91]).

Since the isolation of embryonic stem cells (ESCs) by Thomson and colleagues in 1998 [85] and the establishment of murine-induced pluripotent stem cells (iPSCs) by Yamanaka in 2006 and in 2007 from human fibroblasts [81, 82], cardiovascular researchers started to consider the idea of differentiating pluripotent cells into cardiomyocytes and use those as the best choice for replacement therapy after myocardial infarction. Together with advancement in the protocols to generate CMs populations with high purity [37, 68], the idea of regenerating the heart with this approach became realistic as evidenced by the numerous ongoing clinical trials [15, 43]. The therapy options developed during these years can be divided in two main categories, which are subjects of this review. The first approach is the direct myocardial injection of PSC-derived CMs as single cell suspensions and the second is the delivery of cardiac PSC-derived tissues as cardiospheres or cardiac patches.

The first works describing cardiomyocyte transplantation in healthy and injured hearts [48, 70], revealed the inherent challenges of the method. These limitations, after years of investigations, could be summed as follows:

1. First of all, survival of injected cardiomyocytes in a hostile environment (such as infarcted heart) is meager and affected by many factors. Cardiomyocytes detached from a matrix activate integrin-mediated cell death pathways (also known as anoikis [7]). Moreover, the hypoxic environment is detrimental because of the nutrient starvation and reduced oxygen concentration, ongoing inflammation and associated cytokines and chemokines release activate different cell death pathways such as TNF-alpha-mediated pathway.
2. Immune rejection could occur since the vast majority of the pre-clinical work done so far did not perform autologous iPSC-CMs transplantation, but allo- or xenotransplantations with regimens of high immunosuppression, which will translate in the clinics in lifelong immunosuppression for the patients.
3. In recent years, many studies described the insurgence of arrhythmias upon the engraftment of PSC-CMs, which could be potentially deadly and are now one of the main limitations for the clinical application of this method [14].

This review provides a comprehensive summary of the findings in the field of PSC-CMs cell therapy and PSC-derived cardiac tissues, highlighting the progress and breakthroughs over the past 2 decades and describing the pre-clinical work performed so far with an eye pointed to future clinical applications.

## Improving survival and engraftment of hiPSC-CMs

### Pro-survival cocktails

**Fig. 1:**
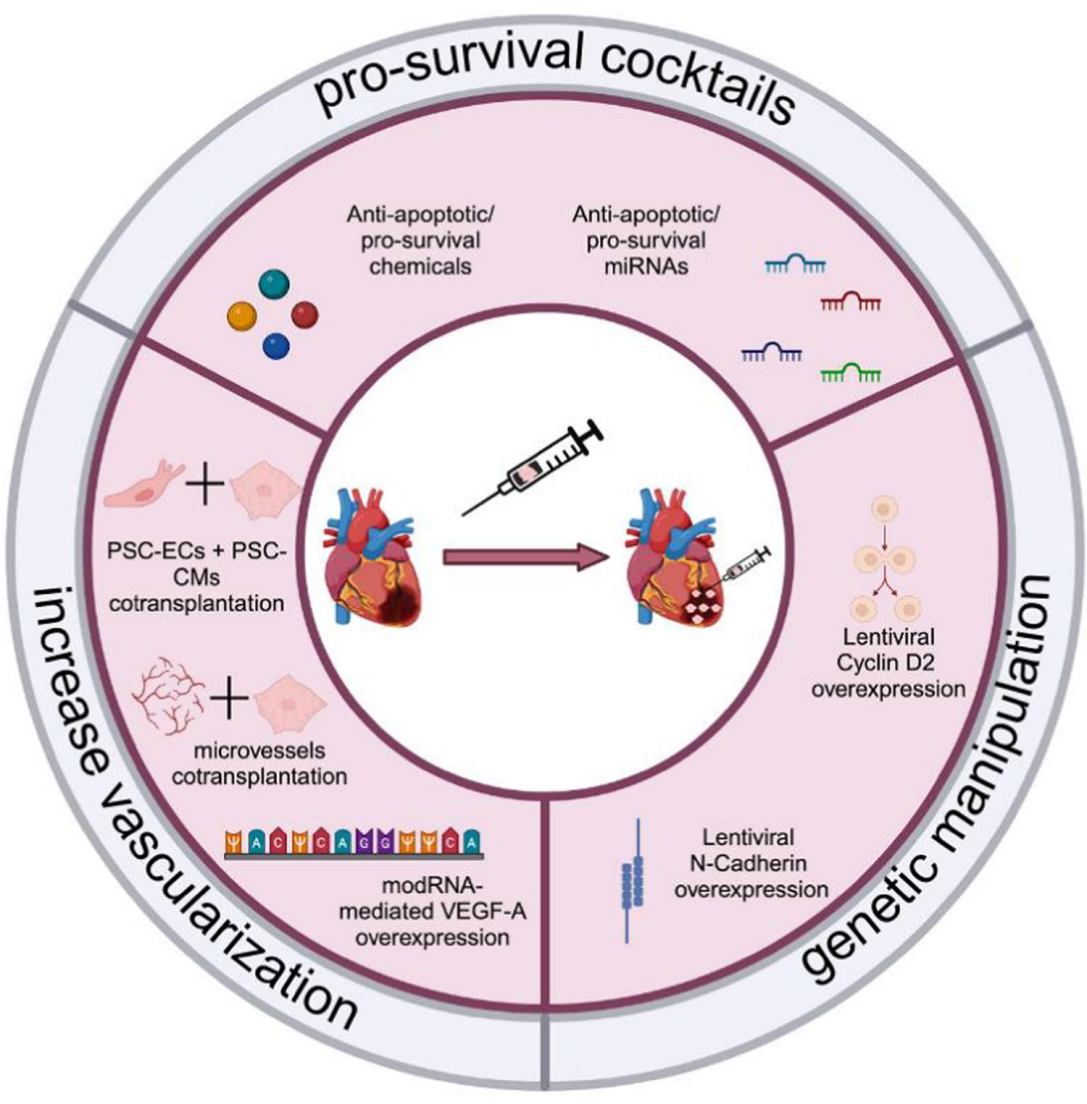
Different approaches to enhance PSC-CMs survival and retention after myocardial injection

The first work aiming at improving PSC-CMs survival after injection was published in 2007 by Laflamme and colleagues [48]. They developed a solution to address the survival and immunological problems of hiPSC-CM therapy. They treated human embryonic stem cell-derived cardiomyocytes (hESC-CMs) with a so-called “pro-survival cocktail” (Fig. 1). This was reported in different studies [5, 10, 54, 73] (with small variations) to enhance cell survival after transplantation. The pro-survival cocktail, as proposed by Laflamme and colleagues, was composed of: reduced growth factors Matrigel (providing a substrate, which prevents cells from undergoing anoikis), ZVAD (pan-caspase inhibitor, preventing cell death), BCL-XL BH4 (BCL-XL recombinant protein, preventing apoptosis), Cyclosporine A (it prevents an infiltrating immune cell invasion into the myocardium from the host), IGF-1 (pro-survival protein), pinacidil (vasodilator, improving the perfusion of the engrafted cells). The pretreatment of hESC-CMs with the pro-survival cocktail showed, in Laflamme’s study, enhanced survival of the cells after transplantation in the infarcted murine hearts, and an improved heart function with increase in fractional shortening and reduced left-ventricular end-diastolic and left-ventricular end-systolic dimensions.

A different approach was, then, used in 2011 by Joseph Wu’s group. Their study showed how miRNAs could play a critical role in improving cell engraftment [30] (Fig. 1). MiRNAs are 18–25 nucleotide long non-coding RNAs, which have been widely investigated in the last decades as main targets for drug development and disease modeling, because of their pivotal role in, virtually, all biological processes through post-transcriptional repression of gene expression [1, 2, 19, 56, 86]. After screening miRNAs, previously reported for having pro-survival or anti-apoptotic functions, they selected miR-21, miR-24, and miR-221 as candidates to increase survival of CMs. They infected mouse-derived cardiovascular progenitor cells (CPCs) with lentiviruses carrying a combination of the aforementioned miRNAs and could show an improvement in survival of the cells and an improvement in fractional shortening in an infarcted SCID mouse model. However, more studies are needed to prove the long-term efficacy and safety of lentiviral delivery of miRNAs cocktails in the injected cells and myocardium. Moreover, upregulation of miRNA-21 is known to induce fibrosis in the myocardium via exosome transfer requiring more in-depth investigation [86].

### Genetic manipulations

Genetic manipulation prior to cell engraftment could be an option to enhance cell survival and integration into the host myocardium, being the rationale for the work published by Zhu and colleagues in 2018 [97]. They generated a cell line overexpressing CCND2 (cyclin D2), a known cell proliferation inducing cyclin, to enhance transplanted CM proliferation during the engraftment (Fig. 1). They could demonstrate that a higher proliferation rate of hiPSC-CMs results in higher engraftment efficiency and higher vascularization in the infarcted mouse hearts. A second study was published by the same group in 2021, investigating the therapeutic efficacy of CCND2 overexpression in hiPSC-CMs, using a large animal model [96]. They demonstrated that cyclin D2 overexpression in hiPSC-CMs was beneficial for the recipient pig hearts improving ejection fraction, preventing post-infarct hypertrophy, and reducing the scar size. They could show that CCND2 overexpression affected not only hiPSC-CMs proliferation but also host CMs and endothelial cells (ECs) proliferation. Mechanistically, they attributed these effects to the modulation of the YAP/TAZ development-related pathway, through miRNAs delivered by extracellular vesicles released by hiPSC-CMs. However, their approach raises some questions about safety since it has been reported that YAP/TAZ pathway is involved in cancer and fibrosis development, increasing the risks associated with its over-activation [25, 36, 63, 92]. While the authors speculate that the shortage of telomeres will eventually lead to a stop in the proliferation rates of the transplanted CMs, more long-term studies are needed to prove the safety of this method.

Again, the same group published a different study in 2020 using genetic manipulation to enhance cell integration and survival after hiPSC-CM transplantation. Here the authors overexpressed the adherens junctions’ component N-Cadherin [55], a protein crucial for maintaining intercalated disks which facilitate the electro-mechanical coupling between cardiomyocytes (Fig. 1). N-cadherin loss results in altered formation of intercalated disks, loss of cell-to-cell interaction, development of dilated cardiomyopathy, and impaired cardiac function [47]. In Lou and colleagues’ study, the lentivirus-mediated overexpression of N-cadherin in hiPSC-CMs before transplantation led to a significant increase in connexin 43 expression, higher engraftment rate, higher vascularization, and smaller infarct size in the engrafted murine hearts. The authors attributed these effects to activating pro-survival and neoangiogenesis pathways. Concerns remain regarding the lentiviral overexpression and the upregulation of connexin 43, leading to higher conduction velocity registered in these cells with possible development of severe arrhythmias that were not further analyzed in this study.

### Increasing vascularization within the graft

One of the main reasons why hiPSC-CMs do not survive the transplantation is the lack of vascularization in the infarcted area, which could lead to a severe hypoxic injury of the cells. Vascularization is a crucial aspect of cell therapy and tissue engineering with promising results not only in cardiovascular research [3, 8, 24] but also in different fields [57, 83]. As shown by Zangi and colleagues in 2013 and Carlsson and colleagues in 2018, a single injection of VEGF-A modified RNA (modRNA) could improve heart function already after 1-week post-infarct by increasing the vascularization in the infarct area and reducing the development of myocardial fibrosis [8, 94]. ModRNAs are RNAs with modified nucleotides incorporated for better transfection efficiency and stability, showing less toxicity and immunogenicity than other gene delivery methods [38, 39, 46, 59]. Zangi et al*.* could also demonstrate that the newly formed vessels from the VEGF-A modRNA injected group were more stable and less leaky than the ones formed by VEGF-A DNA (a plasmid carrying the coding sequence of VEGF), and they could show that this effect was due to the higher and shorter release of the protein [94]. In the cell therapy context, a higher vascularization of the injury site could provide sufficient oxygen and nutrients to the PSC-derived cells to survive and integrate into the host heart. With this idea in mind, Ai and colleagues pretreated hiPSC-CMs with a VEGF-A modRNA (Fig. 1), showing highly promising results in neovascularization, leading to better survival of engrafted cells compared to the control group in an infarcted rat model [3]. Moreover, in their study, the modVEGF-treated group elicited improved heart parameters such as left ventricular ejection fraction (LVEF) and left ventricular fractional shortening compared with the engraftment of iPSC-CMs not treated with VEGF, 4 weeks post-surgery.

A different approach was employed by Sun and colleagues [80] (Fig. 1), using microvessels isolated from adipose tissue and combining them with ESC-CMs to provide early vascularization of the graft to ultimately improve graft survival. They could prove not only improved survival of the transplanted CMs, but also higher fractional shortening of the injected hearts when compared to CM injection alone and CMs combined with dispersed endothelial cells. Furthermore, they have proven how the engrafted microvessels anastomosed with the host cardiac vasculature.

There is more work investigating co-transplanting CMs with another type of cells. In fact, in 2019, Bargehr and colleagues engrafted both, hESC-CMs and hESC-derived epicardial cells (hiESC-EPI) in infarcted athymic rat hearts, showing, again, improvement in heart function, when compared to the ESC-CMs group only [5]. They could demonstrate that their results were mainly due to paracrine signals from hESC-EPI, which led to a higher proliferation in ESC-CMs and higher grade of vascularization.

The next step was made shortly after by Cheng and colleagues, by co-transplanting hiPSC-CMs and hiPSC-derived endothelial cells (hiPSC-EC) both, in mice and non-human primates (NHPs) [10]. They first detected a higher maturity in hiPSC-CM co-cultured with hiPSC-EC, compared to the ones cultured alone. Afterwards, they engrafted hiPSC-CMs alone or in combination with hiPSC-EC in mice and then, in NHPs (in the latter, 28 days post-ischemia–reperfusion injury, as a chronic injury model), showing bigger graft size, improved heart function, and lower arrhythmogenicity in the co-transplantation group as compared to the hiPSC-CM group alone. They attributed these effects to the more extensive neovascularization and higher maturity of the engrafted hiPSC-CMs promoted by the co-transplantation of hiPSC-ECs.

In summary, pro-survival cocktails and genetic manipulations to improve “on-site” revascularization are valid approaches to improve cardiac cell therapy, and a combination of them could probably pave the way toward a clinical application (Fig. 1, Table 1).

**Table 1:** How to improve the survival of transplanted hiPSC-CMs

Rationale	Strategy	Treatment	References
Increasing survival Decreasing apoptosis	Pro-survival cocktails	Anti-apoptotic/pro-survival chemicals Anti-apoptotic/pro-survival miRNAs	[48] [30]
Enhancing CM proliferation/Enhancing integration	Genetic manipulation	Lentiviral overexpression of Cyclin D2 Lentiviral overexpression of N-cadherin	[97] [96] [55]
Increasing oxygen and nutrients support to the grafts	Increasing vascularization	ModVEGF-overexpression Microvessels co-transplantation PSC-EC co-transplantation	[3] [80] [10]

## Cardiac spheroids and cardiac patches

A different approach to enhance CMs survival and retention within the host heart is to create a cardiac microenvironment before transplantation. Different groups explored the possibility of using heart-derived cardiospheres, cardiac spheroids, or cardiac patches to improve the survival of the grafts and cardiac performance upon transplantation.

### Cardiac spheroids

**Fig. 2:**
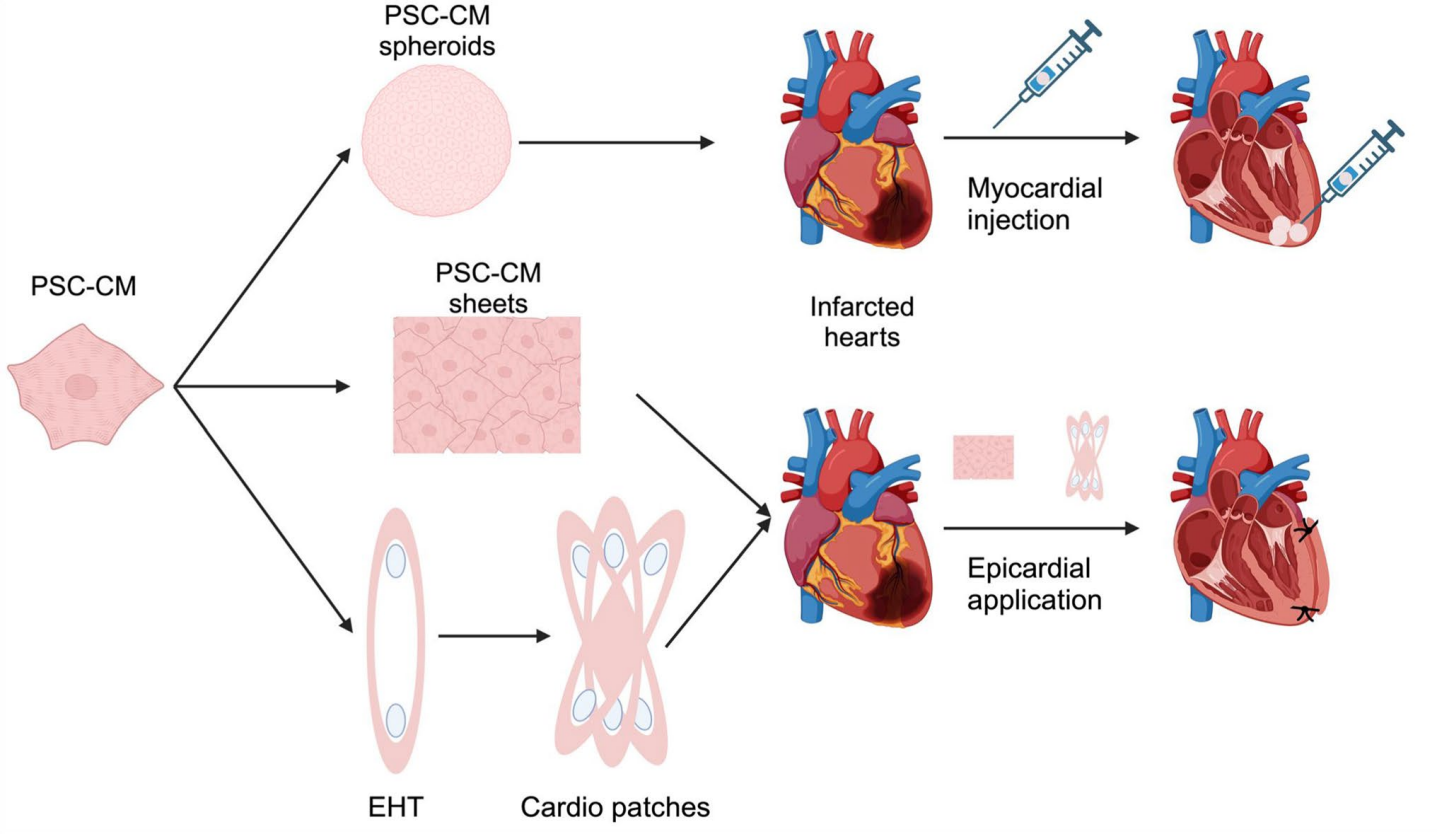
PSC-derived heart tissues and their application in infarcted hearts

Spheroids are assembled by culturing one or multiple cell lineages that spontaneously aggregate to form adherent populations [18, 62]. In 2015, Gallet et al*.* performed a series of *proof-of-concept* experiments, proving that self-assembling cardiospheres derived from minipig hearts were able to remuscularize infarcted pig hearts when infused in coronary arteries, giving benefits in terms of vascularization within the infarct area, decrease in adverse remodeling (less collagen deposition after the ischemic injury), and improvement in heart parameters, such as ejection fraction [21]. Furthermore, they could prove the safety of intracoronary infusion of particles > 45 µm, without obstructing heart vasculature. In 2021, Jun Fujita’s group aimed to use the approach of transplanting cardiac spheroids to increase the survival of the transplanted cells (Fig. 2) [41]. Using cryoinjury as a heart failure model, Fujita and colleagues transplanted cardiac spheroids (composed of a highly pure population of hiPSC-CMs) in immunocompromised rats and pigs, showing larger engraftment size than dispersed CMs and improvement in cardiac function. They observed that after 2 months post-transplantation, both in rats and pigs, the CMs hardly survived in the host hearts. The benefits were, therefore, mechanistically linked to a higher secretion of VEGF from the grafts followed by a higher vascularization. A second study using cardiac spheroids was published in 2024, by the same group [45]. In this study, the authors transplanted two doses of hiPSC-derived cardiac spheroids (hiPSC-CS) for evaluation for later clinical trial (named LAPiS, NCT04945018). They evaluated retention, integration, and maturation, through immunofluorescence staining, demonstrating not only that a higher maturation of the engrafted cells could be achieved faster in cardiac spheroids when compared to other studies using dispersed hiPSC-CMs [52], but also that a lower dose of CMs is sufficient for a clinical setting (probably because of a higher survival and retention of engrafted cells). It is also worth mentioning that this last study was the one that used the highest purity of ventricular CMs (> 99% positive for cTnT), which could explain the higher maturation and lower incidence of ventricular arrhythmias. These two studies paved the way for the use of standardized protocols for hiPSC-CM application that could, in the future, be applied to clinical practice for the treatment of heart failure. However, more studies are needed to investigate in more details different aspects, such as immune rejection and the insurgence of potentially fatal arrhythmias.

### Historical overview of cardiac patches

So far, we have discussed methods to improve the myocardial delivery of cells or spheroids injected into the heart /coronary vessels; however, parallel to this, several groups developed a different approach: laboratory-grown heart tissues that can be applied epicardially (cardiac patches, Fig. 2) [93]. Cardiac patches can be composed of cell sheets [22, 75] or cells combined with different types of scaffolds [22, 49, 98, 99]. The first studies in using non-injection-based cell therapy delivery were published in 2002 and 2006 by Zimmermann and colleagues [98, 99]. Mixing neonatal rat cardiomyocytes with collagen I, Matrigel, and medium in a circular mold, they generated contracting engineered heart tissue (EHT, Fig. 2) and transplanted them in non-infarcted [98] (as a *proof of concept* for feasibility) and infarcted rat hearts [99]. In infarcted rats, they could demonstrate the benefits of cardiac patches in terms of heart performance, but they also showed for the first time the electrical coupling between host and graft and vascularization of the patch. Different works focused on the benefits of the cardiac patches compared to the myocardial injection of dissociated single PSC-CMs, the first one published in 2009 by Hamdi and colleagues [22]. They used two different scaffolds to engraft human myoblasts: one was a fibrin-based scaffold, which was degraded after 9 days and then substituted by a collagen film for the delivery, and one was a collagen sponge (Gelfoam, already used in different context of tissue engineering [66, 67]). They compared the intramyocardially injected myoblasts with the ones which were scaffold-embedded (in a comparable cell density) and applied epicardially, showing for the last a better outcome on heart function, less fibrosis, and higher angiogenesis. Similar results were obtained by Sekine and colleagues in 2011 using neonatal rat cardiomyocyte-derived sheets, obtained by culturing the cells in temperature-sensitive culture dishes and detaching them by cold [75]. They demonstrated that the cell sheets are engrafting more efficiently compared to single cells, translating into a better recovery of the hearts after MI and a better vascularization of the tissue. Similar to the CMs injection, also in the cardiac patch field, an important topic is the vascularization of the transplanted tissue. To achieve a clinically relevant result, the patches used for large mammals and human purposes are expected to be thicker and larger than the ones used for rats and mice, but lacking vasculature, this could affect nutrient and oxygen diffusion to the core of the patch decreasing the survival of the graft [53], creating a necrotic core as already observed in different studies [16, 79]. Different approaches to enhance vascularization within the cardiac patches [53] have been proposed. The first method which has been applied since long time in the field is to mix PSC-CMs with endothelial cells and fibroblasts. Two independent groups reported the advantage of including HUVECs or PSC-EC, necessary to form vessels, and embryonic fibroblasts, necessary to stabilize them [9, 78]. It is notable that the vessels formed in the patches anastomosed with the host when transplanted into rat hearts [78]. Another approach, reported by Noor and colleagues in 2019, is to bioprint a patch with endothelial cells building perfusable vasculature similar to the human host myocardium [65]. Different scaffold-based methods have been reported, describing the use of more porous materials that allow a better diffusion of oxygen, since the size of the pores is important to create an optimal environment for neovasculogenesis [12, 53]. An alternative is to fuse smaller EHTs to increase the tissue size prior to transplantation by creating mesh-like bigger structures (Fig. 2), enhancing the area for oxygen diffusion, method that has already proven itself in remuscularizing animal hearts [69]. The advancements in cardiac patch technology are promising, but still much work is necessary to improve the vascularization of the patches and the standardization of this method.

### Patches today: a hope for heart failure patients of tomorrow

Cardiac patches represent today one of the leading methods in regenerative cardiology investigated for the treatment of heart failure patients. In recent years, many publications showed the possibility of building *bona fide* human myocardium, changing cellular and extracellular patch composition. One of the first steps prior to a clinical application of cardiac patches was the confirmation that they engraft long term in myocardial infarction models and contribute to slowing down remodeling and fibrotic processes as demonstrated by Wu group in 2015 [71]. In this study, Riegler and colleagues engrafted engineered heart tissue in a chronic myocardial infarction rat model, without achieving sufficient functional improvement (probably due to the lack of cell integration by gap junction formation between graft and host) but they could demonstrate two important points: first, it was possible to monitor the graft for a maximum of 220 days, showing a better alignment and maturation of the CMs; second, they showed that the scaffold itself had beneficial effects. Another step moving closer to a clinical application was the standardization of patch casting and composition under good manufacturing practice, performed by the Zimmermann group in 2017 [87]. With their standardized composition (equivalent fractions of CMs: non-cardiomyocytes cultured in Matrigel constructs, in serum-free medium, comprising growth factors such as fibroblast growth factor-2 [FGF-2], insulin-like growth factor 1 [IGF-1], transforming growth factor-β1 [TGF-β1], vascular endothelial growth factor 165 [VEGF_165_]), they could show differentiation characteristics not reported before. The CMs achieved rod shape with well-defined and aligned sarcomeres, with the predominant expression of the ventricular isoform of the myosin light chain (MLC2V), ventricular-like action potential, and a positive force–frequency relationship present only in post-natal hearts. At the end of the study, the authors showed the scalability of their engineered heart myocardium, using custom-made scaffolds to create 15 × 17 mm and 35 × 34 mm patches. Afterward, the same group tested their findings engrafting rhesus EHTs in healthy and infarcted rhesus macaques, which could be detected up to 6 months (endpoint of the study) [34]. In this study, they could show a good dose-dependent enhancement of heart contractility and ejection fraction. On a histological level, they showed vascularization of the EHT at 3 and 6 months, but a relatively immature CM population in the patch, with higher expression of immature isoforms of troponin I and regulatory myosin light chain. These findings and developments are now under investigation in a clinical phase I/II trial (BioVAT-HF, NCT04396899), whose first results are published in the same study [34]. Here, the authors showed the very first human heart treated with cardiac patches, highlighting that the results, in terms of maturation and vascularization, were very similar to the ones observed in monkeys. Regarding the immunological aspects, they could show some infiltration of macrophages and natural killer cells, even though they used quite high immunosuppression levels [34].

A different approach is the one published in 2019 by the Sawa group [40], where they compared major histocompatibility complex (MHC)-matched CMs sheets with MHC-mismatched CMs sheets transplantation in monkeys’ ischemic hearts. They could demonstrate the benefits for transplantation of both groups in terms of reduction in ventricular dilatation and increase in fractional shortening after 3 and 6 months, showing that in their experimental setting, effects from treatments with grafts of both groups were irrespective of MHC matching. The immunological aspects of this work will be discussed in the next section. These results motivated the authors to establish a protocol to bring the therapy to a clinical trial, which is currently ongoing in phase I [42, 61]. They reported for the first time human engraftment of hiPSC-CM patches, with 3 and 6 months as time points for evaluation of safety and function. The three patients reported had suffered from myocardial infarction in the past and had an ejection fraction ≤ 35% at the time of the surgery. The patients received immunosuppressive treatment for 3 months before it was discontinued. Benefits in reduction of heart volumes and increase in ejection fraction 3 and 6 months post-surgery and enhanced coronary blood flow in two out of three cases were reported. Assuming that after 3 months, the engrafted cells would be cleared out by the immune system, as showed before in monkeys [40], the authors postulated that the benefits observed were mainly due to a better vascularization of the infarcted area, rather than increase in force generation resulting from the graft. On a safety level, patients showed no signs of engraftment arrhythmias (EAs, discussed in the next session) or tumor formation. These first results bring new optimism towards the clinical application of not only cardiac patches but all cardiac, PSC-derived therapies.

## Engraftment safety

### Avoiding immune rejection

The immune surveillance from the host immune system could be one of the main drawbacks for PSC-CM therapies. Most of the work described so far have adopted a common strategy of using high doses of different types of immunosuppressive drugs (cyclosporine A [5, 48, 73], methylprednisolone [3, 73], tacrolimus [3]), or immunocompromised animals were used to avoid rejection in rodents [5, 10, 30, 41]. This translates into an immunosuppressive regimen for the patients if this method is translated into the clinical practice, which is already applied concomitantly in other organ transplantation bearing already known risks [72]. In terms of cost/benefits, the idea of having a lifelong regimen of immunosuppressive drugs for a therapy that has not been yet proven a huge benefit to patients’ hearts does seem questionable. Different studies addressed these concerns. Shiba and colleagues, for instance, generated a monkey iPSC cell line homozygous for the major histocompatibility complex (MHC) haplotype HT4, injecting, then the differentiated CMs into cynomologus monkeys heterozygous for HT4 to avoid immune rejection [77]. They could demonstrate that in an immunosuppressive regimen, transplanting MHC-mismatched monkeys triggered a severe T lymphocyte response, leading to immune rejection of the engrafted cells. However, when they transplanted iPSC-CMs in MHC-matched monkeys, they showed no intramyocardial immune infiltration after 12 weeks. Still, it is important to mention that the engrafted monkeys were under an immunosuppressive regimen, even if lower doses of drugs were used as compared to other studies. It is notable that in other studies, a higher dose of immunosuppressive drugs was sufficient to avoid immune rejection of the xenografts [10]. Similar work was done by Kashiyama and colleagues, using CMs sheets [40]. As described above, they transplanted MHC-matched and MHC-mismatched CMs sheets in infarcted monkey hearts to evaluate the function and immunogenic response, treating both groups with immunosuppressive drugs, and evaluating the grafts at 3, 4, and 6 months (longer than the previous work from Shiba and colleagues). The CMs sheets survived up to the fourth month but could not be detected after 6 months, in both transplantation groups, showing an immune rejection for both MHC-matched and MHC-mismatched CMs. However, there was a strong infiltration of CD3-positive lymphocytes after 3 months in the MHC-mismatched group only, while in the MHC-matched group, rejection occurred probably through different mechanisms. They also showed that the withdrawal of immunosuppressive drugs after 3 months post-transplantation did not affect the heart function. Therefore, they proposed that after 4 months, different mechanisms are responsible for the rejection of MHC-matched CMs, then CD3-lymphocytes-invasion and MHC-mismatched CM-line could be probably used safely in clinical settings under immunosuppressive therapy for 2 or 3 months [42, 61]. However, more work is needed in this direction to understand the exact mechanisms behind cell graft rejection in the heart. Recently, Lin et al. described long-term engraftment of autologous iPSC-CMs in two monkeys [52]. This elegant study paves the way to autologous manufacturing and engraftment, eliminating the risk of immune rejection and giving a good insight into in vivo maturation of iPSC-derived cells. In fact, in this study, the authors generated two specific cell lines carrying a construct expressing a sodium/iodide symporter (NIS, usually not expressed by cardiomyocytes), which allowed them to trace in vivo the fate of these cells. Based on NIS expression, they could show that after 8 months, the cells underwent maturation: they were not proliferating anymore, they expressed N-cadherin at the intercalated disks, and they lacked expression of the fetal isoform of myosin regulatory light chain (MLC2a), replaced by the adult ventricular isoform MLC2v. However, at this time point, the engrafted cardiomyocytes still expressed slow skeletal troponin I isoform (ssTnI, fetal isoform), and connexin 43 (Cx43) which was not located at the intercalated disks. These signs of incomplete maturation were not present in the second animal, sacrificed after 14 months. No significant differences were observed between host and engrafted cardiomyocytes in this animal, showing a high maturation of the engrafted cells. Moreover, no immune infiltration or rejection of the cells was evident in both monkeys, indicating that autologous cell transplantation could be one of the best options for translating cell therapy into clinical practice. An alternative to autologous transplantation has been shown by Hu et al., investigating the usage of a hypoimmune cell line, generated by knocking down MHC class I and II molecules and overexpressing CD47 [31]. This cell line could escape immune surveillance when transplanted undifferentiated in muscles and differentiated as pancreatic islets in the pancreas of immunocompetent mice. More studies are needed to evaluate the efficacy of this cell line in the heart. Still, this *proof-of-concept* work fuels optimism toward the generation of a single cell product that could be used for all patients eliminating the manufacturing problems linked to the generation of an autologous cell line specific for each patient.

### History of engraftment arrhythmias (EAs)

**Fig. 3:**
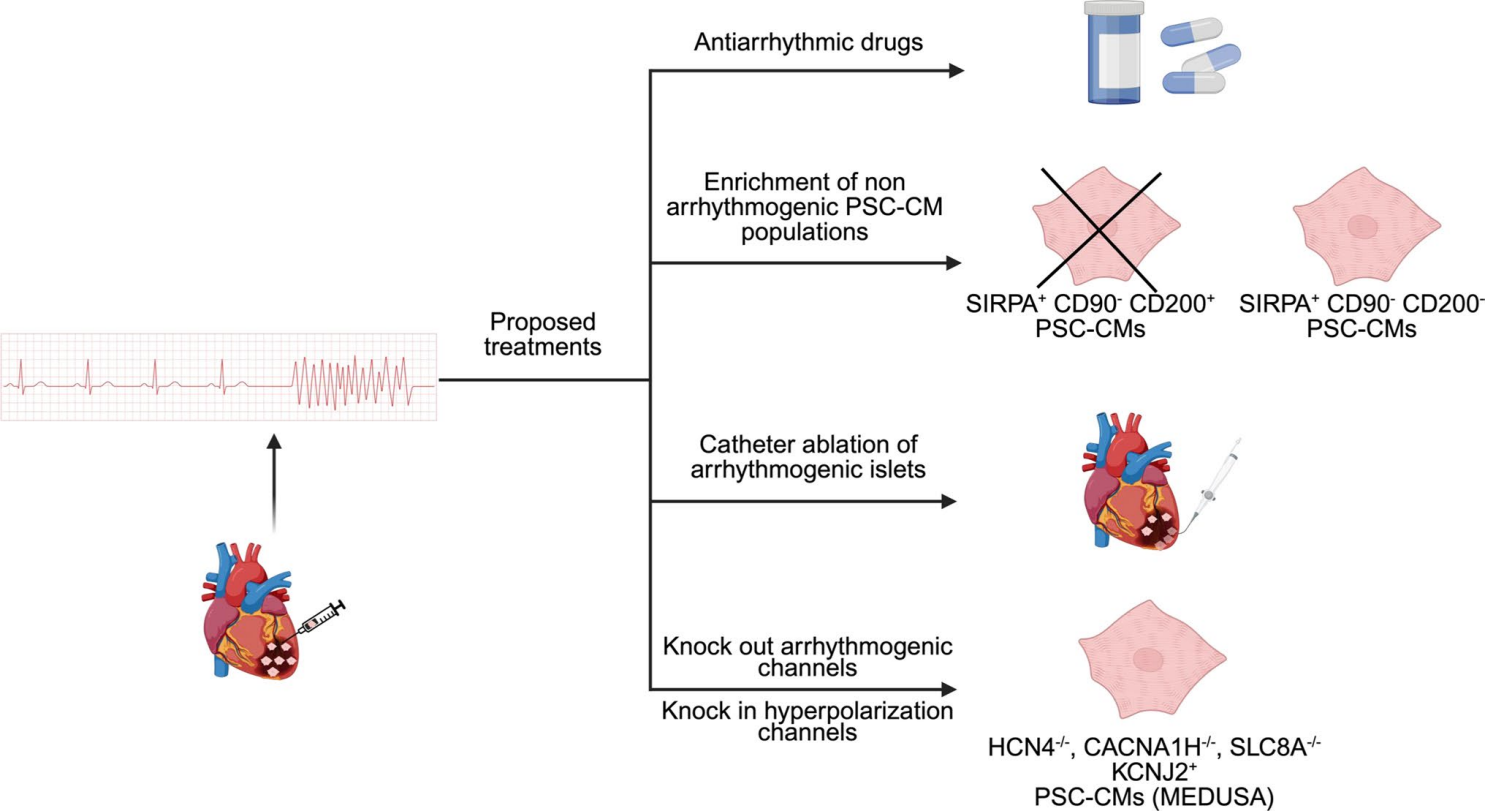
Proposed EAs treatment options

In 2014, Murry’s group engrafted hESC-CMs in infarcted NHP for the first time in a dose-finding study to evaluate the integration and vascularization of the grafts. They, then, noted that all the engrafted monkeys presented with arrhythmias which were absent in the monkeys that did not receive the cell treatment (Fig. 3) [11]. The authors described this type of arrhythmias as “premature beats and runs of ventricular tachycardia”, they also noted wide QRS complexes. The authors speculated about the origin of the so-called engraftment arrhythmias (EAs), which were not observed in small animal models. These differences could be attributed to graft size and heart frequency. Bigger heart sizes, such as in monkeys, require a higher number of cells to be transplanted to observe a significant remuscularization effect. The depolarization wave that propagates within the graft could be more substantially slowed by engrafted cells in a bigger graft than a smaller graft, allowing for re-entry mechanisms. On the other hand, the heart frequency of mice and rats are faster, they outpace the slower rates of hiPSC-CMs, not allowing for generation of automaticity (the ability of PSC-CMs to depolarize spontaneously) or re-entry mechanisms. Similar results were obtained by Shiba and colleagues when transplanting monkey hiPSC-CMs in monkeys’ infarcted hearts [77]. Here, they showed sustained ventricular tachycardia in the transplanted group, that peaked at 14 days and declined afterward, probably due to the higher maturation of the cells after this time point. The mechanism underlying EAs remained unclear for many years, until Murry's group first investigated their origin in 2018 [54]. In this study, they engrafted hESC-CMs in primate hearts to study remuscularization and EAs. At first, they generated a myocardial infarction (MI) model that had a bigger size, because in the previous study, MI was very small to appreciate any difference in heart function (left ventricular ejection fraction of 60% versus 65% at baseline) [11]. Once they injected differentiated hESC-CMs, they showed successful engraftment after 4 weeks and 3 months, with improved heart parameters. The engrafted hearts prior to transplantation had an ejection fraction of 39.4 ± 2.1%, whereas after 4 weeks post-engraftment, it increased to 50.0 ± 1.9%. The main finding of this study, however, was the frequency of arrhythmias. They did not show any significant difference in arrhythmias insurgence between injected and control monkeys, but they elegantly demonstrated via electrical mapping, overdrive pacing, and cardio-version studies the different origins of arrhythmia in the two groups. While, in control groups, the arrhythmias were more likely to originate from re-entry mechanisms due to the big infarct sizes, in the cell-treated group, they showed that the insurgence of arrhythmias had an ectopic impulse generation within the ventricle. These findings were confirmed later by Romagnuolo et al*.* in 2019, using a pig MI model [73]. They performed similar electrophysiological experiments aimed at proving the origin of EAs, showing that the engrafted hiPSC-CMs generated a focal impulse capable of inducing ventricular tachycardia up to 261 bpm ((beats per minute), normal rate of pigs is 90 bpm). They also showed that, after 3 weeks, these arrhythmic events stopped in concomitant with a better cell maturation: higher expression of ventricular isoform of myosin light chain (MLC2v) and switch from fetal troponin I isoform (ssTnI) to the adult cardiac-specific one (cTnI), higher expression and better polarization of connexin 43 (Cx43). This last finding suggests that poor maturation of the cells could lead to spontaneous depolarization and to arrhythmias. Furthermore, in this study, the danger of untreated EAs emerged clearly with one of the tested subjects died because of ventricular tachycardia that degenerated into ventricular fibrillation. For these reasons, it can be assumed that EAs are one of the main obstacles for the translation of hiPSC-CMs cell therapy to humans [14].

Interestingly, most of the studies performed with cardiac patches did not show any signs of arrhythmias [35, 40, 71]. This phenomenon could be explained by two different mechanisms. First, the PSC-CMs in the engineered heart tissue display a more mature phenotype compared to the PSC-CM used for myocardial injection. Therefore, it is possible that the cardiac patches have less automaticity properties. The second aspect regards the electrical coupling of the patches with the host heart. In fact, in the latest studies, also shown by Kashiyama et al., the protective mechanism of cardiac patches could derive from a paracrine effect rather than cell integration and electro-mechanical coupling to the host myocardium [40].

### Treatments approaches and mechanistic insights into EAs

Even without a fully clear mechanism on origin of the EAs, the first approach to treat these arrhythmic events was tested by Nakamura et al. in 2021 [64]. This approach was quite intuitive and screened for seven clinically available anti-arrhythmic drugs in a pig MI model to test which one(s) would be able to reduce the beating frequency in the engrafted hearts. They could show that only class III broad-acting amiodarone and funny current/HCN4 channel antagonist ivabradine were promising in controlling the rhythm and rate of EAs, respectively. Furthermore, they were able to show that the two agents are synergistic, acting with different mechanisms with combination of both sufficient to reduce fatal events in the tested subjects (Fig. 3). Interestingly, 4 weeks of treatment with anti-arrhythmic drugs was enough to control EAs until electrical maturation of grafts developed, and later, no EAs occurred in most subjects (5 out of 7 that completed the study). In the remaining two subjects, the EAs could be controlled.

The same strategy was proposed few years later by Selvakumar et al.in 2024. In this study, the Chong group focused on the mechanistic aspect of EAs using a multi-omic approach. First, they divided the infarcted pig subjects into five groups: vehicle, vehicle + anti-arrhythmic drugs (AA, using amiodarone and ivabradine), PSC-CM, PSC-CM + AA, and sham control. Then they evaluated the heart responses to engraftment and AA, demonstrating that PSC-CMs alone were not sufficient to ameliorate the infarct outcome. Still, in combination with AA, they resulted in improved ejection fraction and left ventricle stroke volume. Furthermore, they confirmed that the application of the previously published combination of amiodarone and ivabradine was enough to suppress EAs. The authors were able to identify, among the PSC-CMs, a sub-cluster of CMs presumably responsible for EAs, using a three-gene signature (SIRPA^+^ CD90^−^ CD200^+^) and one that, on the contrary, was not linked to EA (SIRPA^+^ CD90^−^ CD200^−^). The EA-prone cluster was composed of atrial and pacemaker-like cardiomyocytes, while the second comprised ventricular and trabecular-like CMs (Fig. 3). To validate this finding, they used a differentiation protocol to enrich for the first group and engraft the cells into the pig MI model. This group showed the highest incidence and severity of EAs, with two of three subjects dying due to the EAs and the third being saved only by catheter ablation (CA). This demonstrates that the atrial and pacemaker-like population is likely responsible for the automaticity observed in hiPSC-CMs and EA development [76].

Different solutions have been proposed to overcome the arrhythmia problem as the application of already discussed anti-arrhythmic drugs. In the Selvakumar et al*.* study, they proposed to have protocols that specifically differentiate the not arrhythmogenic PSC-CMs population, or to use catheter ablation [76]. This procedure aimed to specifically remove the graft foci responsible for EAs with a catheter guided by electroanatomic maps to distinguish the anatomical origin of the EAs (Fig. 3). A different approach could be to improve the electrical maturation of hiPSC-CM before transplantation or in vivo as shown by Cheng et al*.* in 2023. Using a combination of hiPSC-EC and hiPSC-CMs, they could demonstrate a lower incidence of arrhythmic events compared to hiPSC-CMs alone [10]. The most recent therapeutic option was proposed by Marchiano et al. in 2024 [58] by modulating the expression of specific ion channels and transporters that are important for ion fluxes and responsible for the automaticity properties of PSC-CMs. They knocked out three genes (HCN4, CACNA1H, and SLC8A) responsible for funny currents and phase 4 spontaneous depolarization. Then they knocked in one channel (KCNJ2) accountable for the inward flux of potassium and hyperpolarization of the cell, calling this quadruple-edited cell line MEDUSA (modification of electrophysiological DNA to understand and suppress arrhythmias, Fig. 3). The MEDUSA cell line did not generate EAs when transplanted into pigs, compared with all the other groups which were single knockouts or knock-ins, where EAs were present. Interestingly, as stated by Selvakumar et al. [76], the three knocked-out channels in Marchiano’s study were also highly abundant in the cells with the arrhythmogenic signature (SIRPA^+^ CD90^−^ CD200^+^). This gives a different view on the field: when differentiating hPSC to CMs, a highly arrhythmogenic population is generated, which expresses multiple channels responsible for automaticity and EAs. By reducing this population with better-defined differentiation protocols or knocking down the channels responsible for the automaticity, it will be possible to reduce and eventually even eliminate the risk of arrhythmias.

### Teratoma risk

It is well-known that PSC have the ability to generate teratomas when transplanted in immunocompromised subjects, an ability that is also used to test the pluripotency of iPSC lines [95]. This could be a hazard for the health of people receiving PSC-based therapies, as demonstrated also by a case of tumor in a patient in 2009 after neural stem cell therapy [4]. However, development of differentiation strategies with highly pure PSC-CMs, yielding > 95% TnT^+^ cells, omitted the risk for tumor formation, strongly suggesting that the method is safe. Even if evidence points out to the safety of the method, some groups suggested inserting suicide genes in the cell lines, to eliminate the cells if teratoma formation is present and the same approach could also be used if arrhythmia risk develops [33].

## Discussion and future perspective

In this review, we discussed how the stem cell therapy field has developed for the treatment of ischemic cardiomyopathy and heart failure. PSC-CMs therapies can remuscularize (at least partially) infarcted hearts. Over the years, scientists developed different strategies, such as the use of pro-survival cocktails, genetic manipulation, co-transplantation strategies, and tissue engineering, to overcome the various challenges they faced to translate this therapy to heart failure patients. Even if more work is needed to unravel the final unknowns, such as the insurgence of EAs and immune rejection mechanisms, we believe the PSC-CM therapy has entered the translation phase into the clinical setting. As testified by the numerous ongoing clinical trials (reviewed elsewhere [43]), we are at a historical moment that can shift the paradigm of heart failure treatment and change patients' lives. Still, a lot of work has to be done not only to understand which are the long-term implications of the therapy, but also to develop differentiation protocols gaining more mature and non-arrhythmogenic cardiomyocytes considering the safety aspects. We can conclude that the perspective of treating patients with PSC-CM is realistic and in the next years, it will be crucial to fully understand its real potential.
